# Cavitary Pulmonary Infarction in a Case of Pulmonary Embolism After Successful Vascular Recanalization

**DOI:** 10.7759/cureus.5983

**Published:** 2019-10-24

**Authors:** Kiran Shivaraj, Erum Zahid

**Affiliations:** 1 Internal Medicine, Brookdale University Hospital and Medical Center, Brooklyn, USA; 2 Pulmonary and Sleep Medicine, Brookdale University Hospital and Medical Center, Brooklyn, USA

**Keywords:** pulmonary embolism, thrombectomy, thrombolysis, pulmonary cavity

## Abstract

Cavity formation after pulmonary embolism can be a result of infarction; however, the data available on the incidence rate were obtained from the cases of patients treated with anticoagulation without recanalization. It is yet unknown if interventions like catheter-directed alteplase or thrombectomy reduce the risk of cavity formation. We present an interesting case of a patient who developed pulmonary cavity and possible secondary infection after successful vascular recanalization with catheter-directed alteplase and thrombectomy.

## Introduction

Pulmonary embolism is one of the known etiologies of pulmonary cavity formation [1]. There are several case reports of a pulmonary cavity developing after a pulmonary embolism, but in all those cases, no intervention was done, and these cases were only treated with anticoagulation. There are no data available on the incidence of pulmonary cavity formation after catheter-directed thrombolysis or thrombectomy. This is a rare case of a patient who presented with cavitary pulmonary infarction in a case of pulmonary embolism, despite undergoing successful vascular recanalization. The premature stoppage of direct-acting oral anticoagulation (DOAC) might have added to the risk of rethrombosis, leading to pulmonary infarction and subsequent cavity formation.

## Case presentation

**Figure 1:**
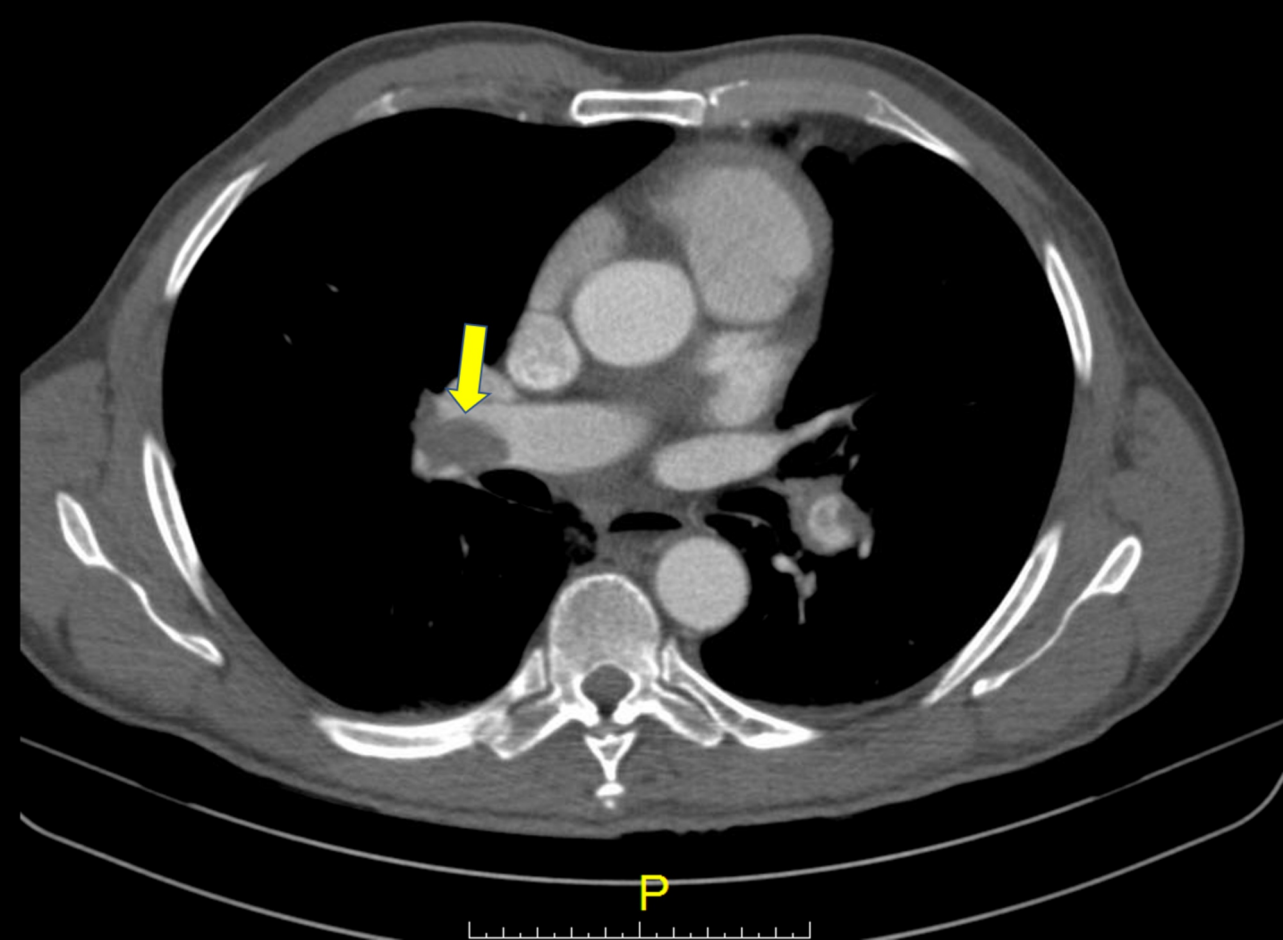
CT angiogram of the chest showing thrombus in the right pulmonary artery (yellow arrow)

A 61-year-old man with no premorbid illness presented to the emergency department with concerns of chest pain and shortness of breath. Upon review of an electrocardiogram (ECG) showing an S1Q3T3 pattern and elevated d-dimer levels, a computerized tomography (CT) angiogram of the chest was performed that showed a moderately sized pulmonary embolism in the pulmonary artery with the right greater than left (Figure 1). Anticoagulation therapy was initiated for the patient, but due to the high Pulmonary Embolism Severity Index score and evidence of right ventricular dilatation with a pulmonary artery systolic pressure (PASP) of 50 to 55 mmHg, the patient was taken to the catheterization laboratory [2]. A catheter-directed infusion of alteplase was performed [3]. After completion of the infusion, anticoagulation therapy with enoxaparin was continued, and there were no complications. Four days later, a repeat ECG was performed, and a persistent right ventricular strain with right ventricular dilation and an increase in PASP to 70 to 75 mmHg were shown. Based on this, a percutaneous thrombectomy of the right pulmonary artery was performed. This has led to successful recanalization of the pulmonary artery and resulted in a significant reduction in pulmonary arterial pressure. The patient was discharged and given DOACs.

**Figure 2:**
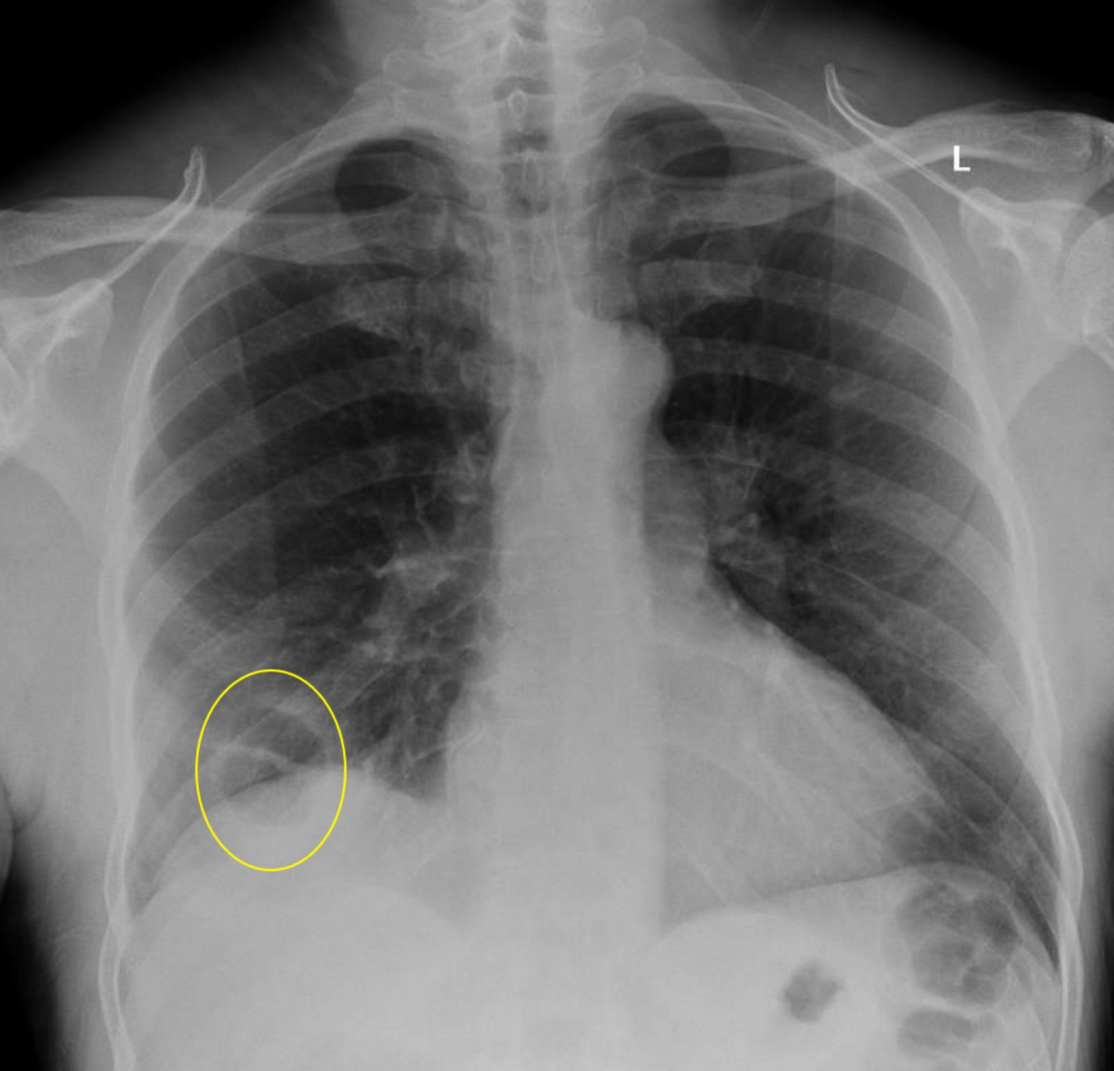
Cavitary lesion visible in the right lower lobe (yellow circle)

**Figure 3:**
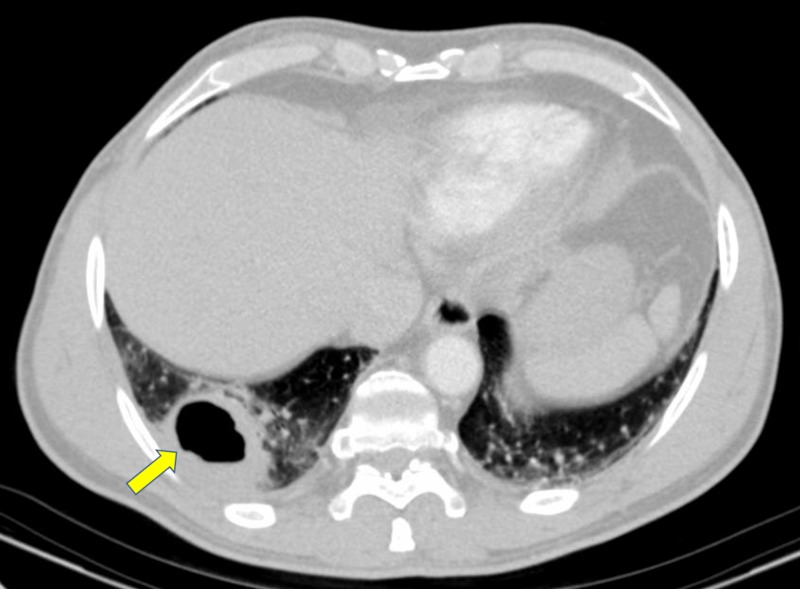
Cavitary mass in the right lower lobe (yellow arrow)

He returned after five weeks with concerns of severe right-sided pleuritic chest pain. On further questioning, he revealed that he had stopped taking the DOAC for one week as his insurance did not cover the medication. A chest x-ray was performed and revealed evidence of an apparent cavity in the posterior right base of the lung measuring 3 cm x 4 cm x 3.6 cm with a thick irregular wall (Figure 2). This finding was not present in the chest x-ray done a few days earlier. A CT angiogram of the chest was repeated that showed resolving bilateral pulmonary emboli and an interval development of a 4.2 cm x 6 cm x 5 cm cavitary mass in the right lower lobe (Figure 3). The patient had no history of fever, recent travel, or loss of weight. To rule out tuberculosis, gamma interferon testing was done; the results were negative, and blood cultures were repeatedly negative for *Mycobacterium tuberculosis*. He was treated with ceftriaxone and metronidazole as a lung abscess could not be ruled out. A subsequent chest x-ray showed an air-fluid level in the cavity, but as the patient was clinically improved, he was discharged with DOAC and educated regarding compliance.

**Figure 4:**
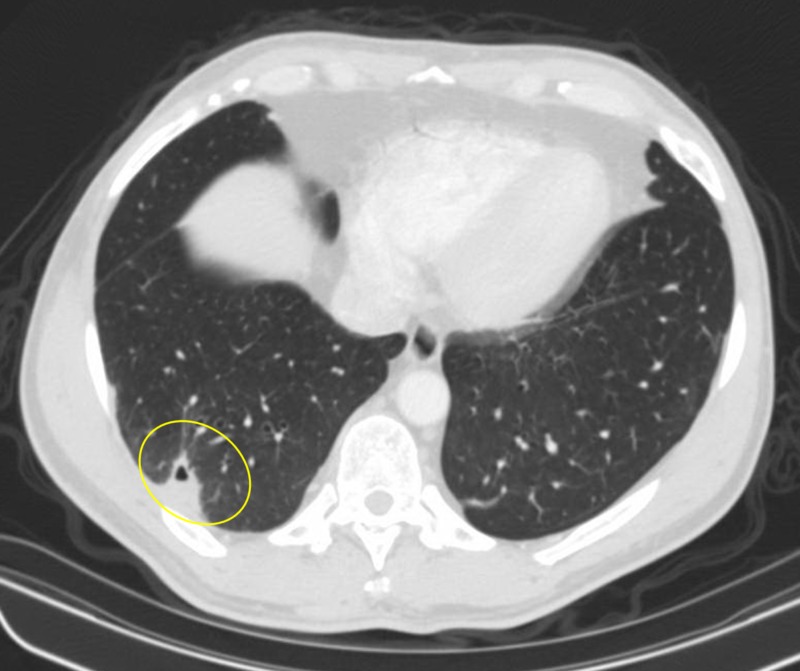
Opacity in the right lower lobe with significantly reduced cavity size (yellow circle)

One month after discharge from the hospital, he presented to the emergency room with pain in the right lower chest but said the pain had significantly reduced in intensity. He claimed to be compliant with the DOAC, and a repeat CT angiogram was performed. The CT showed possible persistent stenosis or partial thrombosis of the right pulmonary artery branch vessel within the right mid-lung; a persistent 2-cm air space opacity within the posterior right lower lobe with a small cavitary component was significantly reduced from what had been seen in the previous CT (Figure 4).

## Discussion

This article presents a patient who presented with a pulmonary cavity after developing a pulmonary embolism. Pulmonary infarction is a known complication after a pulmonary embolism with the incidence being about 10% [4]. The low incidence of pulmonary infarction has been largely been attributed to the dual blood supply of the lungs. Approximately 4% to 7% of the cases of pulmonary infarction subsequently lead to cavity formation [5]. There have been several case reports on cavitary pulmonary infarction after pulmonary embolism, but there are two important features unique to this case. First, this patient had undergone catheter-directed tissue plasminogen activator infusion and a thrombectomy with successful recanalization. Second, this patient prematurely stopped the DOAC. This might have contributed to the increased thrombogenicity leading to rethrombosis [6,7].

The average time for the formation of a cavity after a pulmonary embolism in an infected embolism is 18 days (range, 6 to 40 days) while that in a bland infarct is 28 days (range, 7 to 120 days) [3], making it more likely to be aseptic infarction. Note that the resolution of symptoms with antibiotics brings up the possibility of the cavity being infected. Even so, an infection is more likely to be the consequence rather than the etiology of cavity formation.

There has been no study that has determined the incidence of a pulmonary cavity after successful recanalization for pulmonary embolism. An important question arose through this case: Does recanalization increase the risk of thrombogenicity in the pulmonary vessel? This occurrence was compounded by the sudden withdrawal of DOAC that might have led to thrombosis, pulmonary infarction, and cavity formation. How long the continued risk of thrombosis is and how long should the anticoagulation be continued after thrombectomy are the questions that need to be answered. Patients with idiopathic (unprovoked) thromboembolism have a substantially higher rate of recurrence, corresponding to an annualized event rate of 7.9% per patient-year [8], and hence will benefit from anticoagulation. However, whether anticoagulation also prevents pulmonary infarction has yet to be studied.

## Conclusions

Even though pulmonary cavity formation is known to occur with pulmonary embolism, the extent to which this complication is prevented by modern intervention like catheter-directed thrombolysis and thrombectomy has yet to be studied. Another topic that needs attention is the role of direct-acting anticoagulation in preventing infarction and cavity formation.
